# High Ki-67 labeling index correlates with aggressive clinicopathological features in papillary thyroid carcinoma: a retrospective study

**DOI:** 10.1186/s13044-025-00265-4

**Published:** 2025-11-01

**Authors:** Defi Nurlia Erdian, Maria Francisca Ham, Dina Khoirunnisa, Agnes Stephanie Harahap

**Affiliations:** 1 Department of Anatomical Pathology, Faculty of Medicine, Universitas Indonesia/Dr. Cipto Mangunkusumo Hospital, Jakarta, 10430 Indonesia; 2 Human Cancer Research Center-Indonesian Medical Education and Research Institute, Faculty of Medicine, Universitas Indonesia, Jakarta, 10430 Indonesia

**Keywords:** Papillary thyroid carcinoma, Ki-67, Proliferation index, Metastasis, Invasion, *BRAF*V600E

## Abstract

**Background:**

Papillary thyroid carcinoma (PTC) is the most prevalent thyroid carcinoma that possesses a wide range of biological behavior. Various clinical and histopathological features, including *BRAF*V600E profile, are used to stratify the risk of disease recurrence in thyroid carcinoma. Recent studies have taken an interest in the potential of the proliferation index, Ki-67, in predicting the aggressive nature of PTC.

**Methods:**

This is a single-center, retrospective study that included 92 PTC cases diagnosed at the Department of Anatomical Pathology, Faculty of Medicine/Dr. Cipto Mangunkusumo Hospital, from January 2019-December 2022. Continuous and categorical analysis of Ki-67 labeling index (LI) was studied and compared with various clinicopathological features and *BRAF*V6000E status.

**Results:**

Ki-67 LI was identified in PTCs (range = 0.1–17.1%, median = 2.45%). Higher Ki-67 LI (median value and Ki-67 LI > 5%) is significantly correlated with older age, advanced stage, lymph node metastasis, distant/organ metastasis, aggressive histological subtypes, unencapsulated tumors, lymphovascular invasion, extrathyroidal extension, and *BRAF*V600E mutation.

**Conclusion:**

The proliferation activity shown by Ki-67 LI correlates with aggressive tumor characteristics and may imply possible prognostic significance in diagnosing PTC.

## Introduction

Papillary thyroid carcinoma (PTC) is a differentiated thyroid carcinoma that accounts for 80–90% of the overall thyroid neoplasm [1]. It possesses a wide range of biological behavior, from slow growing to highly aggressive malignancies. PTC often influences women in the third to fourth decade, but prognoses tend to get worse in older patients [2]. According to the American Thyroid Association 2015, thyroid carcinoma can be classified into a low, intermediate, and high-recurrence risk depending on the presence of clinicopathological features such as older age, tumor multifocality, extrathyroidal extension (ETE), lymph- vascular invasion (LVI), lymph node metastases, distant metastasis, and genetic profile of *BRAF*V600E and or *TERT* rearrangement [3]. The World Health Organization (WHO) classification of tumors also recognizes histological subtypes of PTC that are associated with aggressive pathology features and poorer disease prognosis. These subtypes include tall cell, hobnail, columnar, oncocytic, and solid/trabecular [4, 5]. Although PTC generally shows a favorable prognosis, the presence of the aggressive features and certain histological subtypes is known to influence disease prognosis, including the heightened recurrence rate, radioablation resistance, and decreased disease-free survival [3, 6–8].

Ki-67 is an antigen protein located in the nucleus, which reflects cellular proliferation ability. Ki-67 labeling index (Ki-67 LI) has been routinely used as a diagnostic and prognostic indicator in other organ malignancies such as breast [9], prostate [10], and pancreas [11]. Although its role in thyroid neoplasm is still controversial, previous studies have identified Ki-67 LI as a potential diagnostic indicator for PTC [12], and is beneficial to differentiate follicular thyroid carcinoma from other relatively more benign follicular patterned-thyroid tumors, such as adenoma [13]. Limited studies with conflicting findings have also shown the association between Ki-67 expression and invasive tumor characteristics such as LVI, ETE, lymph node metastasis, and distant metastasis [14–16]. Recently, a study by Kakudo et al. has proposed risk classification of thyroid follicular cell carcinomas into low-risk, moderate-risk, high-risk, and undifferentiated carcinoma categories using the Ki-67 LI [17]. The Ki-67 LI is identified as an independent predictor for disease-free life, with elevated values correlating with reduced cause-specific survival [13, 15].

This present study aims to further analyze the correlation between the immunohistochemical expression of Ki-67 with clinico-pathological features of PTC tumors, including the mutational status of *BRAF*V600E. We hypothesize that there is a positive correlation between high Ki-67 expression and aggressive features of PTC tumors, enriching the global understanding of Ki-67 in PTC and its potential as a prognostic indicator.

## Materials and methods

### Ethical clearance

This study has received ethical clearance from the institutional ethical review board through letter number KET-1264/UN2.F1/ETIK/PPM.00.02/2023 and was conducted in accordance with the Helsinki declaration. Informed consent has been waived through letter number ND-762/UN2.FI/ETIK/PPM.00.02/2023.

### Study design and population

This is a retrospective study that included all PTC cases that underwent thyroidectomy and were diagnosed at the Anatomical Pathology Department, Faculty of Medicine, Universitas Indonesia/Cipto Mangunkusumo Hospital from January 2019 until December 2022. The inclusion criteria are cases with accessible clinical data, an available and sufficient number and quality of hematoxylin and eosin (H&E) slides, and formalin-fixed paraffin-embedded (FFPE) tumor tissues from the achieve of the institution. The exclusion criteria include cases with high-grade histological features (high mitotic index and necrosis).

### Clinical and histological features

Clinical data such as age, sex, AJCC clinical stage, tumor size, and the presence of distant organ metastases were collected from the medical records of the patients. Two certified pathologists re-assessed H&E slides of each case for evaluation of histological variables such as histological subtype, the presence of tumor capsule, multifocality, LVI, ETE, and lymph node metastases. Histological subtype of PTC is classified into aggressive and non-aggressive groups. According to the latest WHO classification of endocrine and neuroendocrine tumours, PTC subtypes most consistently linked to aggressive biological behavior are tall cell, hobnail, and infiltrative columnar cell subtypes [5]. In the present study, based on the case distribution within our cohort, the “aggressive subtype” category was defined to include tall cell, solid/trabecular, and oncocytic subtypes. Although not all of these are formally listed as aggressive in the WHO classification, prior literature has reported their association with more aggressive clinicopathologic characteristics [18–22]. The “non-aggressive subtype” category consisted of classic and follicular subtypes. No hobnail or columnar cell cases were observed in this series.

### BRAFV600E mutational analysis

*BRAF*V600E variants were identified in exon 15 of the *BRAF* gene using conventional polymerase chain reaction methods and DNA sanger sequencing analysis, as detailed in previous publication [23].

### Ki-67 immunohistochemical staining

FFPE tissue block from each case is sectioned using a microtome to a thickness of 3–4 μm and placed on glass slides coated with poly-l-lysine. A sequence involving heating at 55–58 degrees Celsius, deparaffinization using xylene, rehydration through a gradual reduction of alcohol concentration, and cleaning with sodium chloride is executed. Endogenous peroxidase blocking is performed using 30% hydrogen peroxide in methanol for 15 min, followed by a 2-minute running water rinse. Antigen retrieval procedure was subsequently performed using Tris-EDTA buffer (pH 9) in a decloaking chamber, at a temperature of 95˚c for 25 min, followed by PBS (pH 7.4) rinsing for 3 min. Background sniper blocking solution (Biocare, CA, US) was added to the sections to block non-specific protein for 30 min, followed by PBS rinsing for 3 min. We performed an incubation process for primary antibody with anti-Ki-67 rabbit monoclonal primary antibody (Roche, Arizona, US) with a dilution ratio 1:50 for 1 h, followed by a 3-minute PBS rinsing. Secondary antibody incubation was performed using Trekie universal link (Biocare, CA, US) for 20 min, followed by TrekAvidin-HRP (Biocare, CA, US) for 20 min. Sections were subsequently incubated with a dilution of diaminobenzidine chromogen buffer for 1 min, followed by water cleansing. Counterstaining was performed using Mayer’s hematoxylin for 10 s, followed by a running water rinse for 3 min. Sections were immersed in 5% lithium carbonate for 10 s, followed by a 3-minute water rinsing. Dehydration with gradual increase of alcohol concentration is performed, followed by xylol cleansing for 3 cycles, each for 5 min. Before covering the glass slide, the mounting solution is added. Sections for negative control were also processed at the same time.

### Ki-67 immunohistochemical evaluation

All stained slides were evaluated blindly by two pathologists under a light microscope (Leica Microsystems, Wetzlar, Germany). The tumors area with the highest Ki-67 expression is identified as a hotspot. Photographs were taken in *≥* 2 high-power fields (40x objective lens magnification) until 2,000 tumor cells were found. The percentage of tumor cells with positive Ki-67 nuclei stained in 2,000 tumor cells was calculated manually by both pathologists. QuPath software was used as a tool to assist in the visualization, selection, and annotation of the cells during manual counting [24]. The interrater reliability between two pathologists was analyzed and showed an interclass correlation coefficient of 0.991 with a 95% confidence interval, which means excellent reliability. For clinical interpretability, Ki-67 LI value was further categorized into two groups- Low and High-based on the predefined cut-off value of 5%. Although previous studies [16, 17, 25] have stratified Ki-67 LI into three categories (< 5%, 5–10%, and > 10%), this study dichotomized Ki-67 LI due to the limited number of cases in the > 10% category. This approach ensured sufficient statistical power and clearer clinical interpretation, while remaining consistent with previously validated cut-offs.

### Statistical analysis

All statistical analyses were conducted using SPSS ver.20 (IBM, NY, USA). The Ki-67 LI was analyzed using both continuous analysis and categorical approaches to provide a comprehensive assessment. In a continuous analysis, the Ki-67 LI values were treated as continuous numerical data. Median, range (min-max), and interquartile range (IQR) were calculated and compared across categories of independent variables. Due to the non-parametric nature of the data, the Mann-Whitney U test or the Kruskal-Walli’s test were applied. The Chi-square or Fisher’s exact test were applied for categorical analysis. A statistically significant result is considered if the *p*-value < 0.05 with a 95% confidence interval.

## Results

This study consists of 92 PTC patients with demographic profiles as detailed in Table 1. This study shows a predominant profile of patients < 55 years old, female, tumor size < 4 cm, early clinical stage, unencapsulated, multifocal tumors, without LVI, and without ETE. Most of the cases show no lymph node metastasis and no distant/organ metastasis. The most common histologic subtype was the tall cells, followed by follicular, classic, solid, and oncocytic subtypes, respectively. We identified 5 cases of papillary microcarcinoma in which tumor size measured *≤* 1 cm.

**Table 1:** Demographic profiles

	Frequency, *n* (percentage, %)
Age (years), median (min-max, IQR)	47.5 (13–75, 22)
< 55	68 (73.9)
*≥* 55	24 (26.1)
Sex	
Woman	69 (75)
Man	23 (25)
Tumors size (cm), median (min-max, IQR)	2.9 (0.7–13; 3.1)
< 4	57 (62)
*≥* 4	35 (38)
Clinical stage	
Stage I-II	81 (88)
Stage III-IV	11 (12)
Encapsulated	
Yes	35 (38)
No	57 (62)
Histological subtype	
Follicular	29 (31.5)
Classic	19 (20.7)
Oncocytic	4 (4.3)
Solid	5 (5.4)
Tall cells	35 (38.0)
Multifocality	
No	18 (19.6)
Yes	74 (80.4)
Lymphovascular invasion	
No	50 (54.3)
Yes	42 (45.7)
Extra thyroidal extension	
No	59 (64.1)
Yes	33 (35.9)
Lymph node metastasis	
No	55 (59.8)
Yes	37 (40.2)
Distant metastasis	
No	77 (83.7)
Yes	15 (16.3)

### Continuous analysis of Ki-67 labeling index

**Fig. 1:**
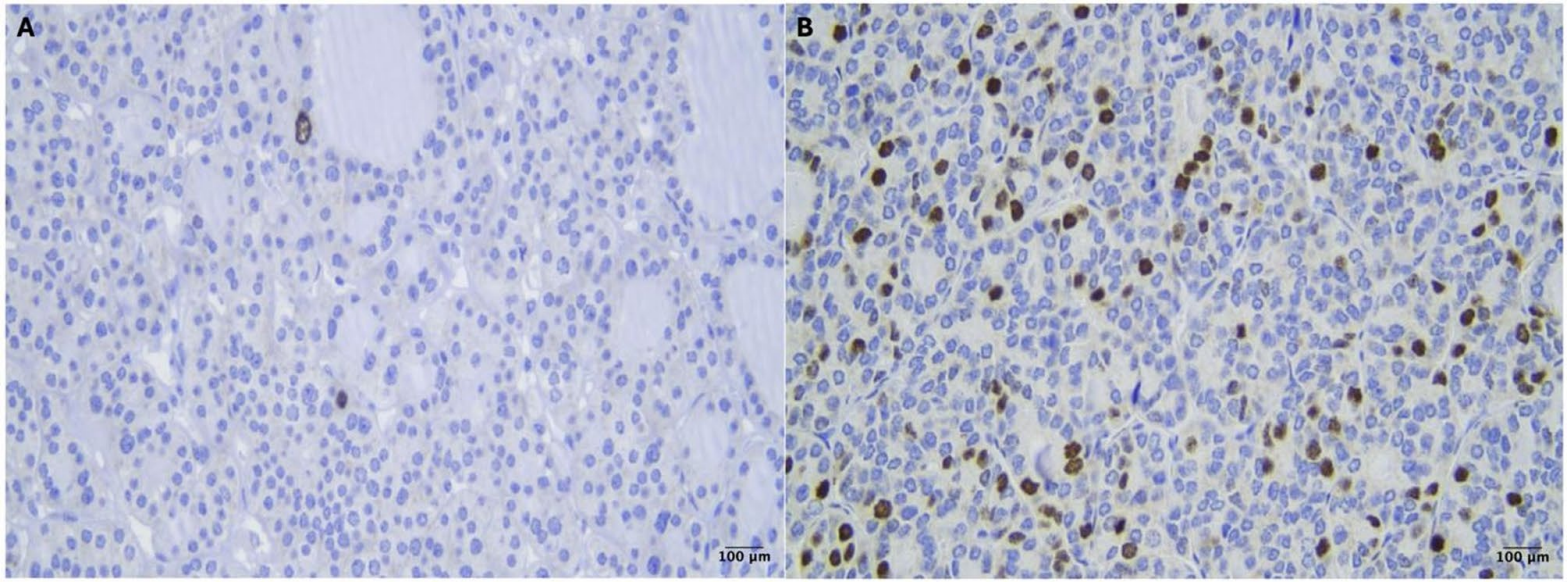
Ki-67 immunohistochemical expression in nuclei of PTC (scale bar 100 μm). (**A**) Ki-67 LI 0.1%. (**B**) Ki-67 LI 15%

Ki-67 LI ranges from 0.1–17.1% with a median of 2.45% and IQR of 2.83. Figure 1 represents the nuclear expression of Ki-67 in PTC tumors. Table 2 shows the different distribution of Ki-67 expression in various clinicopathologic profiles. A higher median of Ki-67 LI is significantly observed in unencapsulated tumors (median = 2.75, *p* = 0.024), advanced (III-IV) clinical stage (median = 5.6, *p* = 0.015), aggressive subtype (median = 3.47, *p* < 0.001), tall cells subtype (median = 4, *p* < 0.001), lymph node metastasis (median = 2.85, *p* = 0.008), distant/organ metastasis (median = 4.45, *p* = 0.014), LVI (median = 3.45, *p* < 0.001), ETE (median = 3.4, *p* < 0.001), and *BRAF*V600E (median = 2.92, *p* = 0.021).

**Table 2:** Correlation between clinicopathological profiles and continuous Ki-67 LI value

	Ki-67 labeling index			
	Median (%)	Min-Max (%)	IQR	*p*-value
Age				
*≥* 55	2.85	0.3–14.9	4.89	0.171*
< 55	2.45	0.1–17.1	2.42	
Sex				
Male	2.45	0.6–17.1	5.45	0.145*
Female	2.45	0.1-13.65	2.83	
Tumors size				
*≥* 4	2.45	0.1–17.1	4.2	0.336*
< 4	2.45	0.3–14.9	2.75	
Encapsulated				
No	2.75	0.25–17.1	2.7	0.024*
Yes	1.75	0.1–9.35	2.4	
Stage				
Advanced (III-IV)	5.6	0.3–14.9	6.9	0.015*
Early (I-II)	2.4	0.1–17.1	2.58	
Histological subtype group				
Aggressive	3.47	0.5–17.1	4.26	< 0.001*
Non-aggressive	1.75	0.1–8.9	1.99	
Histological subtype				
Tall cells	4.0	0.75–17.1	4.00	< 0.001**
Solid	2.65	0.55–14.90	9.48	
Oncocytic	1.95	1.75–2.05	0.23	
Classic	2.5	0.6–5.3	1.85	
Follicular	1.25	0.10–8.90	1.83	
Nodes metastasis				
Present	2.85	0.3–17.1	4.7	0.008*
Absent	2.05	0.1–8.9	2.5	
Distant/organ metastasis				
Present	4.45	0.3–17.1	4.75	0.014*
Absent	2.35	0.1–10.2	2.35	
Lymphovascular invasion				
Present	3.45	0.3–14.9	4.39	< 0.001*
Absent	1.9	0.1–17.1	1.96	
Extrathyroidal extension				
Present	3.4	0.6–17.1	4.65	< 0.001*
Absent	1.9	0.1–10.2	2.55	
Multifocality				
Present	2.55	0.1–17.1	2.53	0.115*
Absent	1.65	0.3–8.9	2.83	
*BRAF*V600E mutation				
Mutated	2.92	0.1–17.1	3.69	0.021*
Wild type	2.15	0.1–14.9	2.4	

*Mann-Whitney U test, **Kruskal-Wallis test. IQR = interquartile range

### Categorical analysis of Ki-67 labeling index

We further categorize the expression of Ki-67 into two different groups- High and Low- based on the cutoff value of 5%. Most patients (75 of 92 patients) displayed low expression of Ki-67 (Ki-67 LI *≤* 5%). Table 3 presents the distribution of low and high Ki-67 LI across various clinicopathological features. Significant associations were identified between Ki-67 LI > 5% and factors such as older age [OR (95% CI) = 4.5 (1.5–13.6), *p* = 0.012], advanced stage [OR (95% CI) = 7.6 (1.9–29.3), *p* = 0.004], aggressive histological subtype [OR (95% CI) = 11.8 (2.5–55.8), *p* < 0.001], lymph node metastasis [OR (95% CI) = 4.8 (1.5–15.1), *p* = 0.005], distant/organ metastasis [OR (95% CI) = 4 (1.2–13.4), *p* = 0.03], LVI [OR (95% CI) = 13.3 (2.8–62.7), *p* < 0.001], ETE [OR (95% CI) = 6.2 (1.9–19.6) *p* = 0.001], and *BRAF*V600E mutation [OR (95% CI) = 4.1 (1.2–13.9), *p* = 0.016].

**Table 3:** Correlation between clinicopathological profiles and dichotomic value of Ki-67 immunohistochemistry expression

	Ki-67 labeling index		OR (95% CI)	*P*-value
	Low (*≤* 5) *n* (%)	High (> 5) *n* (%)		
Age				
*≥* 55	15 (20)	9 (52.9)	4.5 (1.5–13.6)	0.012**
< 55	60 (80)	8 (47.1)		
Sex				
Male	16 (21.3)	7 (41.2)	2.6 (0.8–7.8)	0.12**
Female	59 (78.7)	10 (58.8)		
Tumors size				
*≥* 4	25 (33.3)	10 (58.8)	2.8 (0.9–8.4)	0.051*
< 4	50 (66.7)	7 (41.2)		
Encapsulated				
No	45 (60)	12 (70.6)	1.6 (0.5–5.1)	0.417*
Yes	30 (40)	5 (29.4)		
Stage				
Advanced (III-IV)	5 (6.7)	6 (35.3)	7.6 (1.9–29.3)	0.004**
Early (I-II)	70 (93.3)	11 (64.7)		
Histological subtype group				
Aggressive	29 (38.7)	15 (88.2)	11.8 (2.5–55.8)	< 0.001*
Non-aggressive	46 (61.3)	2 (11.8)		
Nodes metastasis				
Present	25 (33.3)	12 (70.6)	4.8 (1.5–15.1)	0.005*
Absent	50 (66.7)	5 (29.4)		
Distant/organ metastasis				
Present	9 (12)	6 (35.3)	4 (1.2–13.4)	0.03**
Absent	66 (88)	11 (64.7)		
Lymphovascular invasion				
Present	27 (36)	15 (88.2)	13.3 (2.8–62.7)	< 0.001*
Absent	48 (64)	2 (11.8)		
Extrathyroidal extension				
Present	21 (28)	12 (70.6)	6.2 (1.9–19.6)	0.001*
Absent	54 (72)	5 (29.4)		
Multifocality				
Present	61 (81.3)	13 (76.5)	0.7 (0.2–2.6)	0.736**
Absent	14 (18.7)	4 (23.5)		
*BRAF*V600E mutation				
Mutated	33 (44)	13 (76.5)	4.1 (1.2–13.9)	0.016*
Wild type	42 (56)	4 (23.5)		

*Chi-square test. **Fisher’s exact test

## Discussion

PTC, the most prevalent type of thyroid neoplasm, generally has a favorable prognosis. However, there is approximately 2–5% of PTC with aggressive features associated with a high recurrence rate and poor disease prognosis [26, 27]. Although still controversial, previous studies have emphasized the potential prognostic value of Ki-67 LI in the new thyroid carcinoma risk stratification, emphasizing the 5% cut off value which can classify tumors into low-, moderate-, and high-risk carcinoma [16, 17, 25]. This present study supports the finding that elevated expression of Ki-67 is correlated with several aggressive clinical and pathological features, including non-encapsulated tumors, advanced clinical stage, lymph node metastasis, aggressive histological subtypes, LVI, ETE, distant metastasis, and *BRAF*V600E mutation.

Ki-67 is a nuclear antigen that reflects the proliferation capacity of tumor cells. Ki-67 was first described in Hodgkin lymphoma, where the expression is higher in the active cellular life cycle and undetectable in the resting (G0) cellular cycle. Among 92 PTC cases enrolled in the study, the median value of Ki-67 LI is 2.45%, ranging from 0.1 to 17.1%. The value is similar to the findings of prior studies [15, 28, 29]. Intra-tumoral heterogeneity of Ki-67 expression can significantly impact hotspot evaluation, as variability in proliferative activity across different tumor regions may lead to inconsistent LI values. This heterogeneity introduces potential sampling bias and may limit the reproducibility and accuracy of Ki-67 assessment. Prior studies have emphasized the need for standardized protocols to improve consistency and ensure that hotspot regions are truly representative of the tumor’s proliferative behavior.

Prior studies have reported older age as an independent risk factor for disease recurrence in thyroid carcinoma [30, 31]. This present study displayed that older (*≥* 55 years) patient slightly have a higher value of Ki-67 LI (median, 2.85% vs. 2.45%). On categorical analysis, we further found a significant association between older age and Ki-67 LI greater than 5%. This finding aligns with those presented in prior studies [15, 32].

Previous meta-analysis has demonstrated that overexpression of Ki-67 is significantly associated with tumor node metastases stages and further worse disease-free survival in thyroid cancer patients [32]. A study by Mussig et al. has also specifically identified that expression of Ki-67 is positively correlated with the tumor staging in well-differentiated thyroid cancer [33]. These findings corroborate with our study in which we found PTC patients with advanced stage (III-IV) are associated with higher (median, 5.6% vs. 2.4%) expression of Ki-67 LI, as well as Ki-67 LI greater than 5%.

This present study identified that those PTC cases with positive lymph node metastasis showed a higher value of Ki-67 LI than those without node metastasis (median, 2.8% vs. 2%). Furthermore, we also found a significant association between Ki-67 LI > 5% and the presence of node metastasis. These findings corroborate with prior studies [14, 32, 34]. Similarly, Lindfors et al. also identified that Ki-67 greater than 3% is associated with lymph node metastasis [35].

This study identified 15 patients with distant organ metastases at presentation. Among these, the value of Ki-67 LI is significantly higher (median, 4.45%) than those without distant organ metastases (median, 2.25%). This aligns with prior reports [15, 32] and may reflect Kjellman et al. study, in which patients with higher Ki-67 LI are correlated with developing local and distant metastasis at the follow-up [29] These findings may suggest that tumors with higher proliferative activity are more prone to metastasize to lymph nodes or distant organs.

In contrast to the previous studies, which demonstrated a significant correlation between elevated Ki-67 and tumor size above 4 cm [16, 32], our present study revealed no significant differences. Tang et al. showed that a higher Ki-67 labeling index was related to thyroid nodules larger than 1 cm in 571 cases of PTC [12], while Aydogan et al. found that a higher average Ki-67 value was associated with tumors bigger than 1 cm in differentiated thyroid cancers [36]. The disparities may be attributed to a variation in sample size among studies and the different thresholds of tumor size for surgical management in thyroid carcinoma employed in different countries.

The fifth edition of the WHO tumor classification recognizes several aggressive histological subtypes of PTC, including tall cell, infiltrative columnar, and hobnail subtypes [5]. In the present study, the aggressive histological group comprised tall cell, solid/trabecular and oncocytic subtypes, while the non-aggressive group included classic and follicular subtypes, consistent with prior studies [18–21]. Columnar cell and hobnail subtypes were not observed in this cohort, in line with their reported rarity, with an estimated incidences of approximately 0.15–0.4% and 0.3%, respectively [22, 37] This study corroborates previous research [14, 38] by demonstrating that elevated Ki-67 LI is significantly correlated to aggressive subtypes of PTC, with the tall cell subtype exhibiting the highest median of Ki-67 LI, followed by solid, and oncocytic subtypes, respectively. Radi et al. reported a mean value for Ki-67 LI 5.42% (SD 4.06) in aggressive subtype of PTC [38] while our present study found a median Ki-67 of 3.47% (IQR 4.26) in the aggressive subtype groups. Categorical analysis in this study further displayed a significant association between aggressive histological subtypes and tumors with Ki-67 LI greater than 5%. These aggressive subtypes possess unique clinicopathological and molecular characteristics often associated with rapid growth rate, infiltrative features, early metastasis, and high recurrence rate. The elevated proliferation rate shown by Ki-67 LI in these subtypes may signify and corroborate the tumor’s aggressive characteristics [16].

The presence of tumor capsule in thyroid pathology is important and is correlated with clinical behavior and prognosis [39]. This present study found a significant association between unencapsulated PTC and higher Ki-67 LI (median, 2.75% vs. 1.75%). The interaction of unencapsulated tumors with the surrounding stromal and immune microenvironment may enhance the secretion of growth factors and cytokines, further promoting tumor cell proliferation. Encapsulated follicular variant of PTC is known to show indolent behavior and has been reclassified under the new entity of encapsulated non-invasive follicular variant of PTC to avoid overtreatment and unnecessary surgery [40]. Further findings also emphasized that regardless of the presence of invasive or high-grade cytological features, poorly differentiated thyroid carcinoma tumors that still maintain even a minimal fibrous capsule is associated with better overall survival rate than those unencapsulated ones [41].

This study also identified the significant differences in Ki-67 LI between PTC tumors with LVI and those without. Among PTC with LVI, the value of Ki-67 LI was higher compared to those without (median, 3.45% vs. 1.9%). We also found a significant association between Ki-67 LI greater than 5% and the presence of LVI. Similar to the role of tumor capsule, the assessment for tumor invasion in thyroid pathology is crucial to determine disease entity and further patient prognosis. Chowdhury et al. has previously shown an association between LVI and Ki-67 LI [14]. Earlier research has also established that the presence of LVI correlates with aggressive tumor characteristics and an increased incidence of disease recurrence [42, 43] This may elucidate the correlation between tumor proliferation activity, as indicated by Ki-67 LI, and LVI features.

In addition, this study also analyzes the significant association between ETE and Ki-67 LI greater than 5%. ETE is defined as tumor invasion to the surrounding tissues including neck muscle structures, soft tissue, or other organs, and is also the important prognostic feature in thyroid carcinoma. Among 33 patients with positive ETE, the value of Ki-67 LI is significantly higher (median, 3.4%) than those without ETE (median, 1.9%). The higher proliferation index displayed in the tumors with ETE supports prior findings that identified that ETE is associated with nodal and distant metastasis, as well as poor overall survival [15, 32, 44]. A recent study by Masui et al. emphasized the potential combination of the degree of tumor extension and Ki-67 LI for the recurrence-risk stratification in PTC [25]. However, conflicting findings was as seen in prior studies, in which the correlation between Ki-67 intensity and ETE was not found [12, 14]. The discrepancy might be attributed to the different immunohistochemical staining and evaluation of Ki-67 LI. As stated previously [25], the average and hot spot count for evaluating Ki-67 is incomparable and might yielded different value of Ki-7 LI. A standardized counting for Ki-67 in a tumor area with the most proliferating activity (hot spot) is needed to minimize the evaluation discrepancies.

*BRAF*V600E is the most prevalent driver gene mutation in PTC and is associated with aggressive features of the tumor and poorer disease prognosis. Although not routinely done in clinical settings, molecular evaluation for *BRAF*V600E or *TERT* mutation may complement the prognostic strength of other clinical and histological features in thyroid risk stratification. Aligning with prior study [45, 46], we found a significantly higher Ki-67 expression in tumors with *BRAF*V600E mutation compared to the wild type. In this context, Ki-67 LI may serve as a practical and accessible surrogate marker of tumor aggressiveness, especially in resource-limited settings where molecular testing is not routinely available. While further validation is needed, these findings support the potential value of incorporating Ki-67 into routine histopathological evaluation to enhance prognostic stratification alongside molecular and clinicopathologic markers.

This study found that the sex profile of the PTC patients did not exhibit a significant correlation with Ki-67 LI. A similar study showed the same result [12]. This may occur because although sex is important factor in PTC epidemiology and prognosis aspects, its impact on the cellular proliferation is minimal, considering the Ki-67 expression is more closely related to intrinsic tumor characteristics [47] Although the multifocality in PTC tumors is known to have prognostic value for predicting disease recurrence rate [48], our finding corroborates prior studies [12, 36], in which we found no substantial differences in Ki-67 expression across different multifocality statuses of the tumors.

The limitations of this study include its retrospective design and relatively small sample size. Although significant associations were identified between several variables, the findings should be interpreted with caution, as some statistical tests produced wide confidence intervals. Another limitation of this study is the lack of data for any disease outcome, such as recurrence and disease-free survival rate. Lastly, the inherent subjectivity of Ki-67 immunohistochemical evaluation and the intra-tumoral heterogeneity of its expression represent methodological limitations of this study. Variability in proliferative activity across different tumor regions may affect the accuracy and reproducibility of hotspot-based LI measurements. Although interobserver reliability was evaluated in this study, intraobserver variability was not assessed due to practical limitations. However, the distinct nuclear localization of Ki-67 staining, as opposed to cytoplasmic markers, typically results in minimal intraobserver variability. A large prospective study with a more standardized Ki-67 evaluation protocol that follows the disease outcome is also needed to validate the actual prognostic role of Ki-67 immunohistochemical in PTC.

## Conclusions

The proliferation activity of the tumor cells shown by Ki-67 LI significantly correlates with aggressive characteristics of PTC, including, an older age, advanced stage, lymph node metastasis, distant/organ metastasis, aggressive histological subtypes, unencapsulated tumors, LVI, ETE, and *BRAF*V600E mutation. Consistent to prior studies, this may imply possible prognostic significance of Ki-67 in PTC diagnosis.

## Data Availability

The datasets used and/or analysed during the current study are available from the corresponding author on reasonable request.

## Abbreviations

ETE: Extrathyroidal extension
FFPE: Formalin-fixed paraffin-embedded
H&E: Hematoxylin and eosin
IQR: Interquartile range
LI: Labeling index
LVI: Lymphovascular invasion
PTC: Papillary thyroid carcinoma
WHO: World Health Organization
